# Health Information Systems’ Support for Management and Changing Work: Survey Study Among Physicians

**DOI:** 10.2196/65913

**Published:** 2025-06-26

**Authors:** Tarja Heponiemi, Lotta Virtanen, Emma Kainiemi, Petra Saukkonen, Jarmo Reponen, Tinja Lääveri

**Affiliations:** 1 Department of Healthcare and Social Welfare, Finnish Institute for Health and Welfare, P.O. Box 30, Helsinki, 00271, Finland, 358 295247434; 2 Research Unit of Health Sciences and Technology, University of Oulu, Oulu, Finland; 3 Medical Research Centre Oulu, Wellbeing Services County of North Ostrobothnia and the University of Oulu, Oulu, Finland; 4 Department of Computer Science, School of Science, Aalto University, Espoo, Finland; 5 Infectious Diseases, Inflammation Center, University of Helsinki and Helsinki University Hospital, Helsinki, Finland

**Keywords:** health care digitalization, leadership position, work change, digitalization, health information systems, support, management, leadership, Finland, cross sectional, online survey, survey, patient information, physician leaders, monitoring, information system

## Abstract

**Background:**

The digitalization of health care has advanced significantly in recent years. Consequently, physicians have needed to increasingly adopt new digital health technologies such as electronic health record systems and other health information systems. Digitalization has changed physicians’ clinical work, work environment, management work, and use of tools for leadership. Many physician leaders have been critical of the capabilities of health information systems (HISs) to support leadership, management, and knowledge management.

**Objective:**

We aimed to examine the association between leadership position and perceived changes in clinical work due to digitalization among a nationally representative sample of Finnish physicians and physician leaders. In addition, we examined physician leaders’ perceptions of HISs as a support for management and whether their opinions differed based on their perceptions on changes in clinical work due to digitalization.

**Methods:**

Altogether 4630 Finnish physicians (2960/4586, 64% women) responded to a cross-sectional nation-wide web-based survey conducted in spring 2021. Perceptions of improved preventive work, facilitated access to patient information, progressed interprofessional collaboration, and accelerated clinical encounters were used as measures of changes due to digitalization. First, we examined with multivariable logistic regression analyses whether being in a leadership position was associated with perceived changes in work due to digitalization (improved preventive work, facilitated access to patient information, progressed interprofessional collaboration, and accelerated clinical encounters in separate analyses) in the total sample. Second, we examined with analyses of covariance whether the variables related to perceived changes in work due to digitalization were associated with perceived management support from HISs among those who had administrative or management responsibilities (n=817). All analyses were adjusted for gender, age, and sector.

**Results:**

Physician leaders had greater odds of agreeing that digitalization had improved preventive work (odds ratio [OR] 1.62, 95% CI 1.33‐1.98), facilitated access to patient information (OR 1.28, 95% CI 1.09‐1.51), progressed interprofessional collaboration (OR 1.81, 95% CI 1.53‐2.14), and accelerated clinical encounters (OR 1.31, 95% CI 1.01‐1.70) than those in nonleadership positions. Furthermore, leaders who perceived these changes in work due to digitalization positively also considered that health information systems supported their management work.

**Conclusions:**

Physician leaders appeared to view the changes in work due to digitalization more positively than other physicians. In addition, those leaders who perceived these changes positively also perceived that HISs supported their management work. Thus, leaders should thoroughly evaluate and address physicians’ perceptions of their routine clinical work and its evolving nature. Doing so ensures access to up-to-date and accurate insights, enabling more effective planning of staffing, training programs, and future implementations. Furthermore, our results show that to guarantee positive views about digitalization among physician leaders, information systems should also support managerial work. This highlights the need to focus on the quality, utility, and usability of information systems.

## Introduction

Digitalization of health care has progressed during the past 20 years. Consequently, physicians have needed to increasingly adopt new digital health technologies (DHTs), such as electronic health record systems (EHRs) and other health information systems (HISs), remote monitoring, artificial intelligence, and robotics [1,2]. The clinical goals of healthcare digitalization include, for example, enhancing the possibilities for preventive work, facilitating access to patient data, progressing with interprofessional collaboration, and improving the efficiency of clinical encounters [3,4].

Digitalization has changed physicians’ clinical work and work environment [5,6], and use of tools for leadership [7]. For example, clinical decision support systems may help physicians by acting as diagnostic support, assisting in administrative functions, helping clinical management, and improving patient safety [8]. Furthermore, physicians have described that the digitalization may support collaboration, decision making, continuous learning, and staying informed [6]. Indeed, health care professionals have stated that increased digitalization has accelerated their work and strengthened multidisciplinary collaboration, due to lower threshold for contacting colleagues of different professional groups [5]. Common data archives and databases facilitate the transfer of information between different units and service providers, and digitization of referrals has expedited the handling of patient matters [5]. Furthermore, previous studies show that HISs can facilitate in filtering, integrating, and interpreting information from diverse sources through better information sharing, improved communication, and timely access to data [9,10].

Despite the identified benefits, digitalization may also lead to pitfalls such as fragmented workflow, alert fatigue, and interoperability [8]. In addition, increasing number of studies report HISs-related stress among physicians [11,12]. Consequently, only a few physicians have perceived that the changes in clinical work due to digitalization were in line with the strategic goals [13], instead, they feel burdened by the undesired changes [14]. A qualitative study suggested that frontline health and social care professionals may perceive more adverse changes associated with digitalization in their work than their leaders [5]. These negative ramifications of digitalization present a challenge, as the reactions of employees play a pivotal role in determining the likelihood of the success or failure of such changes [15,16].

Many physician leaders have yet been critical of the capabilities of HIS support for leadership, management, and knowledge management [17]. They experience a need to put a lot of effort to gathering and updating necessary information from many different sources, and the information may be unreliable [17-19]. Physician leaders working in hospitals have reported that HISs supported decision-making and improved the ease of access to information, but did not improve the speed of access to information [20]. Furthermore, they had to use several systems to support their decision-making and wished for one HIS offering all the necessary information to support their decision-making [20]. Middle management has expressed difficulties in collection, mining, and systematic use of information due to different ways of producing and reporting information of the HISs [19]. Nevertheless, data collection for these findings occurred before the COVID-19 pandemic, during which information assets for knowledge management in healthcare may have evolved.

This study focused on examining the association between leadership position and perceived changes in clinical work due to digitalization (improved preventive work, facilitated access to patient information, progressed interprofessional collaboration, and accelerated clinical encounters) among a nationally representative sample of Finnish physicians and physician leaders. In addition, we examined physician leaders’ perceptions of HISs as a support for management and whether their opinions differed based on their perceptions on changes in clinical work due to digitalization.

## Methods

### Sample

The data were collected with a web-based survey between January and March 2021 as a part of the “EHR systems as a tool for physicians 2021” study [21]. An invitation to participate was sent by email to all working-age (aged 65 y and younger) physicians from the Finnish Medical Association’s register. A detailed description of the data collection, measures used in the questionnaire and the evolution of the questionnaire can be found elsewhere [21]. Altogether 4683 physicians (estimated response rate 24.5%) responded to the survey. We excluded 53 respondents who indicated that they did not use EHRs at all. Thus, the final sample included 4630 physicians. The sample was representative of the target population, with a slightly more older physicians, specialists, and hospital physicians compared to the eligible population [22].

### Ethical Considerations

The study was conducted according to national laws, regulations, and the Declaration of Helsinki. The survey did not require a statement from the ethics committee, given that the Finnish National Board on Research Integrity [23] has defined that the administration of surveys to gather respondents’ opinions, which are not anticipated to result in harm, does not necessitate a statement from the ethics committee. All respondents provided informed consent to participate, participated voluntarily, and received written information about the study. All responses were anonymous and the research data did not include any personally identifiable information. No compensation was provided to participants.

### Measures

The variables used in this study can be found in Multimedia Appendix 1.

Perceived changes in clinical work due to digitalization were measured by respondents’ opinions on the following statements on how the digitalization of health care had changed their work in the past 3 years:

1. Possibilities for preventive work have improved (improved possibilities for preventive work).
2. It has become easier to obtain information on patients (facilitated access to patient information).
3. Interprofessional collaboration has progressed (progressed interprofessional collaboration).
4. Consultations with patients have become faster (accelerated clinical encounters).

The statements were rated: 1=fully agree, 2=somewhat agree, 3=neither agree nor disagree, 4=somewhat disagree, and 5=fully disagree, which were then recoded as 0=disagree or neutral (response options 3‐5) and 1=agree (1-2). These statements were developed by clinicians and healthcare digitalization experts according to the strategic goals of digitalization of health care [4] and pilot tested with physicians. The statements have been associated, for example, with work-related time pressure and stress related to information systems [14].

Perceived management support from HISs was assessed with 3 items (Cronbach α=0.81) measuring the supportiveness of information systems for knowledge management. The respondents were directed to this section if their professional title indicated that they might have a leading or managerial position. Furthermore, the respondents were advised that if they do not have administrative or management responsibilities, they should not grade these statements (817 responded to these statements). They were asked how, in all, do the information systems used in their organization work as tools for leadership and management The items were: “I use information systems daily for activity monitoring,” “information systems facilitate monitoring the quality of activities,” and “information systems help me to monitor the targets set by my unit (eg, numbers of patients, periods of treatment, and types of operations).” The response options with 5-point Likert scale ranged from 1=fully disagree to 5=fully agree. A mean of these three items was calculated, higher scores indicating better support for management. Respondents were advised not to answer this question if they do not have administrative or management responsibilities; thus, those who answered this question were considered as being in leadership position.

Furthermore, the survey asked respondents’ age (which was categorized as under 35, 35‐44, 45‐54, and 55‐64 years) and employment sector (hospitals, primary care, private, and other such as universities). Respondents’ gender was received from registers.

### Statistical Analyses

First, we examined whether being in a leadership position was associated with perceived changes in work due to digitalization in the total sample. Multivariable logistic regression analyses were conducted with the perceived changes in clinical work due to digitalization (improved preventive work, facilitated access to patient information, progressed interprofessional collaboration, and accelerated clinical encounters in separate analyses) as dependent variables and leadership position (yes or no), gender, age, and sector as independent variables.

Second, we examined whether the variables related to perceived changes in work due to digitalization were associated with perceived management support from HISs among those who had administrative or management responsibilities (n=817). Analyses of covariance were conducted, with perceived management support from HIS as the dependent variable and the four statements regarding perceived changes in clinical work due to digitalization as independent variables (in the same analysis), adjusted for gender, age, and sector. All analyses were conducted using IBM SPSS Statistics software (version 28).

## Results

Characteristics of the study sample can be seen in Table 1. Almost 2 in 3 of the respondents were women. More than half of the respondents worked in specialized care. Approximately 1 in 6 worked in a leadership position. In addition, 1 in 5 agreed that digitalization had improved preventive work and more than 2 in 5 agreed that it had facilitated access to patient information. In total, 30% agreed that digitalization had progressed interprofessional collaboration, whereas only 1 in 10 agreed that it had accelerated clinical encounters. Among physician leaders, the mean score for perceiving HISs as supporting management was 2.70 (SD 0.99).

**Table 1.** Characteristics of the study sample.

Characteristics	Value, n (%)	
Gender		
Man	1626 (35.5)	
Woman	2960 (64.5)	
Age (years)		
<35	948 (20.6)	
35‐44	1211 (26.4)	
45‐54	1148 (25.0)	
55‐64	1284 (28.0)	
Sector		
Hospital	2759 (59.6)	
Primary care	1037 (22.4)	
Private	610 (13.2)	
Other	221 (4.8)	
Leadership position		
No	3813 (82.4)	
Yes	817 (17.6)	
Improved preventive work		
Disagree or neutral	3622 (80.4)	
Agree	884 (19.6)	
Facilitated access to patient information		
Disagree or neutral	2565 (56.4)	
Agree	1986 (43.6)	
Progressed interprofessional collaboration		
Disagree or neutral	2948 (65.0)	
Agree	1586 (35.0)	
Accelerated clinical encounters		
Disagree or neutral	4084 (90.2)	
Agree	445 (9.8)	
Information systems as support for management (range 1‐5), mean (SD)	2.70 (0.99)	

Table 2 shows the results of the logistic regression analysis for changes in clinical work due to digitalization in the total sample. Women had lower odds of agreeing that digitalization had accelerated clinical encounters than men. Older respondents had lower odds of agreeing that digitalization had improved preventive work and greater odds of agreeing that digitalization had facilitated access to information than younger respondents. Compared to respondents working in public specialized care, those working in public primary care and private care had greater odds of agreeing that digitalization had improved preventive work and facilitated access to information. Furthermore, respondents working in public primary care had greater odds of agreeing that digitalization had progressed interprofessional collaboration, and respondents working in private sector had greater odds of agreeing that digitalization had accelerated clinical encounters than those working in public specialized care. Respondents in leadership positions had greater odds of agreeing with all the statements concerning clinical work change due to digitalization than others. The clearest difference was observed regarding progressed interprofessional collaboration and improved preventive work.

**Table 2.** The results of the logistic regression analysis for changes in work due to digitalization, shown in odds ratios (ORs) and their 95% CIs (n=4630).

	Preventive work			Access to information			Interprof^a^ collaboration			Accelerated encounters		
	OR (95% CI)		*P* value	OR (95% CI)		*P* value	OR (95% CI)		*P* value	OR (95% CI)		*P* value
Gender			.05			.06			.25			<.001
Man	1^b^			1			1			1		
Woman	0.86 (0.73-1.00)			0.88 (0.78-1.00)			0.93 (0.81‐1.06)			0.65 (0.53-.79)		
Age			.006			.047			.22			.46
<35	1			1			1			1		
35‐44	0.72 (0.57‐0.90)			1.01 (0.84‐1.21)			0.83 (0.69‐1.00)			0.99 (0.72‐1.36)		
45‐54	0.79 (0.63‐1.00)			1.06 (0.88‐1.28)			0.87 (0.71‐1.05)			1.20 (0.87‐1.66)		
55‐64	0.68 (0.54‐0.86)			1.24 (1.03‐1.50)			0.92 (0.76‐1.11)			1.17 (0.85‐1.61)		
Sector			<.001			<.001			<.001			<.001
Hospital	1			1			1			1		
Primary care	1.70 (1.41‐2.05)			1.52 (1.31‐1.77)			1.53 (1.31‐1.78)			1.17 (0.90‐1.52)		
Private	2.88 (2.32‐3.57)			2.06 (1.71‐2.49)			1.17 (0.96‐1.43)			2.19 (1.68‐2.87)		
Other	2.00 (1.43‐2.80)			1.75 (1.32‐2.32)			1.38 (1.03‐1.85)			1.34 (0.85‐2.13)		
Leadership position			<.001			.003			<.001			.04
No	1			1			1			1		
Yes	1.62 (1.33‐.98)			1.28 (1.09‐.51)			1.81 (1.53-2.14)		<.001	1.31 (1.01‐.70)		

a Interprof: interprofessional.

b Reference category.

Table 3 shows the results of the analyses of variance for perceived management support from HISs among those who were in leadership positions. HISs were least experienced as supporting management in public primary care. Multimedia Appendix 2 shows that those who agreed that digitalization had improved preventive work, facilitated access to patient information, progressed interprofessional collaboration, or accelerated clinical encounters perceived HISs as more supportive compared to those who disagreed or were neutral about these statements.

**Table 3.** Results of the analyses of variance for information systems as support for management and its estimated marginal means according to independent variables (n=817).

Variable	*F* test (*df*)	*P* value	Mean^a^ (SE)	
Gender	0.93 (1)	.34		
Man			3.02 (0.08)	
Woman			2.95 (0.07)	
Age (years)	0.54 (2)	.58		
<45			2.94 (0.10)	
45‐54			3.03 (0.08)	
55‐64			2.99 (0.08)	
Sector	9.95 (3)	<.001		
Hospital			2.94 (0.06)	
Primary care			2.61 (0.09)	
Private			3.40 (0.13)	
Other			2.99 (0.16)	
Improved preventive work	6.07 (1)	.01		
Disagree or neutral			2.89 (0.07)	
Agree			3.09 (0.09)	
Facilitated access to patient information	19.01 (1)	<.001		
Disagree or neutral			2.84 (0.08)	
Agree			3.14 (0.08)	
Progressed interprofessional collaboration	23.74 (1)	<.001		
Disagree or neutral			2.81 (0.08)	
Agree			3.16 (0.07)	
Accelerated clinical encounters	13.31 (1)	<.001		
Disagree or neutral			2.80 (0.06)	
Agree			3.18 (0.11)	

a Estimated marginal mean adjusted for all variables in the table.

## Discussion

### Principal Findings and Comparison With Previous Work

This study found that physician leaders agreed more often that digitalization had improved preventive work, facilitated access to patient information, progressed interprofessional collaboration, and accelerated clinical encounters compared to those without leadership positions. This suggests that leaders have a more positive view related to ramifications of digitalization than physicians without leadership positions. However, physician leaders did not experience that HISs support their management work, given that their average rating for support was 2.7 on a scale of 1 to 5. Nevertheless, although physician leaders considered HISs support for leadership and management insufficient, those who agreed with these statements also gave more positive responses about the impacts of digitalization.

Our finding that physician leaders had a more positive view related to digitalization than other physicians is congruent with a previous study [5]. A qualitative study among health and social care professionals suggests that leaders may overlook the new digital skills needs and problems caused by combining continuous learning with busy work schedules [5]. A possible reason why health care leaders may have a more positive attitude toward digitalization is that they play an active role in leading these initiatives by striving to demonstrate a visible commitment towards new DHTs [24]; they also try to convince reluctant users to envision digitalization in a more favorable manner [25]. It has been suggested that the successful implementation of DHTs among health care professionals is contingent upon managers being convinced of its positive effects [26].

Our study found that those leaders who agreed with the positive statements related to the changes in clinical work due to digitalization also perceived more often that HISs supported management work compared to those who disagreed or were neutral. Due to the cross-sectional nature of our study, we cannot identify the causality of this association; that is, we cannot definitely identify whether effective support from HISs leads to improved efficiency in management work for leaders and contributes to a positive view toward digitalization. Alternatively, it could be that those who have noticed that digitalization has improved preventive work, facilitated access to patient information, promoted collaboration, and accelerated clinical encounters are therefore also more inclined to rate that HISs support their management work. It is possible that the general satisfaction with the benefits of digitalization for leaders in health care is reflected in their responses. Thus, it seems important to ensure that leaders keep themselves well-informed about the realities of clinical work and thereby avoid having excessively positive view related to digitalization [26]. Many physician leaders participate also to clinical work which makes it easier to stay up to date about the situation in clinical care. However, physicians working in nonleadership positions need to communicate work-related issues and digital challenges to management, providing nuanced insights for decision-making [26].

Our results suggest sector-specific variations, with physicians working in specialized care perceiving less improvement in preventive work and facilitated access to information compared to physicians working elsewhere. Whereas primary care focuses on preventive measures and health promotion, specialized care physicians mainly treat the diseases in their own specialty [27]. A previous study among specialized care physicians showed that day-to-day operations management HISs supported decision-making to some extent but did not improve access to information [18]. Hospital physicians have also reported that they need to use a number of information systems to support decision making instead of one HIS which they would prefer [18]. Hospital physicians have also reported that they were more able to monitor the use of personnel, equipment, or room resources through HISs compared to primary care physicians [17]. Furthermore, physicians in specialized care settings have previously stated fragmentation of information and data overload resulting from difficult-to-use EHRs [28]. In addition, the implementation of HISs has led to changes in professionals’ workflow, potentially causing cognitive burden [29]. Notably, high levels of stress related to HISs have been reported, particularly among physicians working in specialized care [12]. These challenges may contribute to the perception of less improvement in access to information among physicians in specialized care. Furthermore, contextual factors such as workload, resource allocation and availability, and on-call work may have affected these sector-specific differences.

Physician leaders in primary care perceived that HISs supported less their management work than leaders from other sectors. This is congruent with previous results in Finland which showed that, of different working sectors, leaders from primary care were most dissatisfied with HISs in guiding daily activity, facilitating measurement of functional quality, and monitoring the achieving of the set targets [17]. Primary care leaders have been found to use less managerial HISs compared to physicians in specialized care and private sector [17]. Compared to other sectors, primary care differs in aspects such as care processes, outcome goals, and the validity of summative quality scorecards [30]; thus, needs for managerial HISs also vary. For example, patient-centered reporting and outcomes (eg, reduction in days of avoidable disability) have been suggested as priorities for quality management in primary care [30].

In our study, older physicians were less likely to perceive that digitalization has improved preventive work or facilitated access to information than younger physicians. This corresponds with previous findings that, compared to younger physicians, older physicians are less likely to perceive that HISs will improve data and care coordination [31]. Older physicians have also been found to perceive less affinity toward digitalization, as well as use and trust mobile apps less in their professional lives than younger physicians [32]. Our finding that older physicians did not consider digitalization as having facilitated preventive work, may influence their likelihood to recommend preventive DHTs to patients. Consequently, this may impact the extent of digital preventive work, as patients’ unawareness of the potential DHTs or their benefits has been mentioned amongst the barriers of digital prevention [33].

Our study did not find many gender differences. However, we found that women were less likely to perceive that digitalization had accelerated clinical encounters compared to men. A previous study among physician students did not find gender differences related to fear of digital challenges, but women rated their knowledge in different fields of digital medicine as worse than men, whereas men frequently reported keeping themselves informed about digital medicine outside of their studies [34]. It has been found that women physicians tend to perceive digitalization-related differences in working conditions as more stress-reducing than men physicians [35].

### Limitations

There are some limitations to this study. A major limitation of our study is the use of cross-sectional survey data, thus we cannot draw any causal inferences. Therefore, according to our data it is not possible to say whether efficient support from HISs to management work leads to more positive views toward digitalization or vice versa. Thus, future studies with longitudinal design should examine more thoroughly the causal associations between HISs support for management and perceptions of digitalization. Furthermore, a question related to problems associated with an inflation of the strengths of relationships and with common method variance always arise when using self-rated measures. Self-reported data are subject to many biases such as recall, desirability, and response biases along with problems in accuracy and generalizability [36]. The reliability of the variable measuring how HISs support management was .81; thus, it can be considered reliable.

In addition, even though we adjusted our analyses for age, gender, and employment sector, we cannot put aside the possibility of residual confounding. Some variables that were not adjusted for, such as competence in knowledge management, may have influenced the associations we examined and, thus, cause bias to our results. Furthermore, usability of HISs and experience as an HIS users are among factors that might have affected our findings, for example, by influencing the overall attitude toward HISs. We excluded 53 respondents who did not use EHRs at all; thus, their opinion was not considered here. This was done because we reasoned that their work did not involve such tasks that they could accurately answer our questions related to digitalization and the supportiveness of information systems for knowledge management.

Our results can be considered generalizable to other countries with universal access to health care and long history of HISs [37]. Furthermore, generalizability of our findings is compromised by rather low response rate of our study, which indicates likelihood of nonresponse bias. However, our sample was rather large and it has been shown to be representative of the target population [22]. The response rates have been found similarly low also in other physician studies [38]. Unfortunately, it seems that response rates among health and social care professionals especially in Finland seem to have decreased. A potential factor contributing to the declining response rates may be survey fatigue, given the high volume of various surveys conducted in this sector in Finland. Another reason for low response rate in our survey may be that the survey was conducted via email, which may have caused problems in message deliveries and email addresses might have changed.

### Conclusions

This study found that physician leaders were more optimistic related to the changes in clinical work due to digitalization compared to other physicians. Furthermore, those leaders who perceived these changes positively also perceived that HISs supported their management work. Leaders should comprehensively assess and address the perceptions of physicians in routine clinical work concerning work and its changes. This approach ensures that leaders possess current and accurate information to optimize planning for personnel resources, training initiatives, and future DHT implementations.

Furthermore, to ensure that leaders have a positive view related to digitalization, it is important that HISs support their management work. Thus, it is important to focus on the quality and usability of information systems. For example, it has been suggested that HISs designed for leaders should be flexible and consider the needs of different users to support a shared situational awareness and operational intelligence in the management [39]. It is important that the success of HIS development would be regularly monitored and that future studies would address HISs’ effect to clinical and management work. Especially, because our study was cross-sectional, future studies with longitudinal study design are needed.

## Supplementary material

10.2196/65913 Multimedia Appendix 1 The items of the variables used in this study.

10.2196/65913 Multimedia Appendix 2 The estimated marginal means of information systems’ support for management according to perceived changes in work due to digitalization (adjusted for age, gender, employment sector, and all perceived changes in work due to digitalization variables).

## Abbreviations

DHT: digital health technology
EHR: electronic health record
HIS: health information system
OR: odds ratio
