# Supplementary material for: Health Information Systems’ Support for Management and Changing Work: Survey Study Among Physicians
Source: JMIR Med Inform. 2025 Jun 26;13:e65913. doi: 10.2196/65913 (PMC12226959; doi:10.2196/65913)
Supplement: Multimedia Appendix 1 [file medinform-v13-e65913-s001.docx]

**Multimedia appendix 1.**

The items of the variables used in the present study

**Perceived changes in clinical work due to digitalization**

Electronic health care services have increased. EHR systems have been used for a long time but, in addition, patients are increasingly often offered electronic services, such as self-care services, teleconsultation, appointment scheduling, and services for recording and viewing their data. How has such digitalization of health care affected your work? Please assess the change during the past three years:

1. Possibilities for preventive work have improved (*improved possibilities for preventive work*).
2. It has become easier to obtain information on patients (*facilitated access to patient information*).
3. Interprofessional collaboration has progressed (*progressed interprofessional collaboration*).
4. Consultations with patients have become faster (*accelerated clinical encounters*).

Response options:

1. Fully agree
2. Somewhat agree
3. Neither agree nor disagree
4. Somewhat disagree
5. Fully disagree

**Perceived management support from HISs**

How, in all, do the information systems used in an organization work as a management tool?

*INSTRUCTIONS FOR RESPONDING: If you do NOT have administrative/management responsibilities, please do not grade the statements below; move to the following page.*

1. *I use information systems daily for activity monitoring.*
2. *Information systems facilitate monitoring the quality of activities.*
3. *Information systems help me to monitor the targets set by my unit (e.g., numbers of patients, periods of treatment, types of operations).*

Response options:

1. Fully agree
2. Somewhat agree
3. Neither agree nor disagree
4. Somewhat disagree
5. Fully disagree

**Age:**

Year of birth was asked and according to the year of birth, the age was calculated and categorized as:

1. Under 35
2. 35–44
3. 45–54
4. 55–64

**Main employment sector**

1. Municipality
2. State
3. Private (incl. The Social Insurance Institution of Finland (Kela))
4. Other such as Universities

**Gender** was received from the registers
