# Supplementary figures and images for: Health Information Systems’ Support for Management and Changing Work: Survey Study Among Physicians
Source: JMIR Med Inform. 2025 Jun 26;13:e65913. doi: 10.2196/65913 (PMC12226959; doi:10.2196/65913)

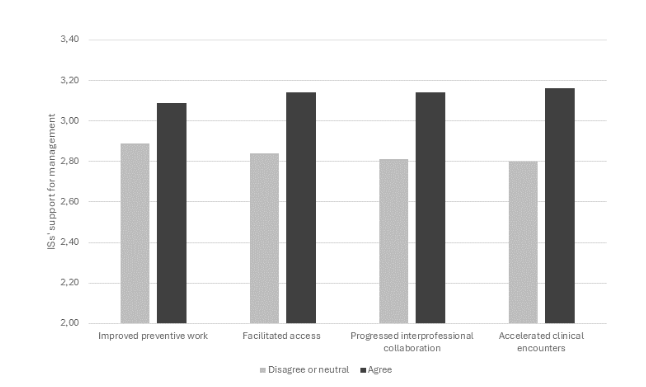

Supplement: Multimedia Appendix 2 [file medinform-v13-e65913-s002.png]
